# Combined immune checkpoint blockade and radiotherapy induces durable remission in relapsed natural killer/T-cell lymphoma: a case report and review of the literature

**DOI:** 10.1186/s13256-021-02798-2

**Published:** 2021-04-30

**Authors:** Elizabeth McGehee, Hetalkumari Patel, Caroline Pearson, Keri Clements, Jesse Manuel Jaso, Weina Chen, Alexandra Callan, Neil Desai, Praveen Ramakrishnan Geethakumari

**Affiliations:** 1 Division of Hematologic Malignancies and Stem Cell Transplantation, Harold. C. Simmons Comprehensive Cancer Center, University of Texas Southwestern Medical Center, Dallas, TX 75390 USA; 2 Department of Hemato-Pathology, Harold. C. Simmons Comprehensive Cancer Center, University of Texas Southwestern Medical Center, Dallas, TX 75390 USA; 3 Department of Orthopedic Oncology, Harold. C. Simmons Comprehensive Cancer Center, University of Texas Southwestern Medical Center, Dallas, TX 75390 USA; 4 Department of Radiation Oncology, Harold. C. Simmons Comprehensive Cancer Center, University of Texas Southwestern Medical Center, Dallas, TX 75390 USA

**Keywords:** Extranodal NK/T-cell lymphoma (ENKL), Immune checkpoint blockade, Radiation therapy, Pseudoprogression, PD-1, PD-L1

## Abstract

**Background:**

Extranodal natural killer/T-cell lymphoma is a rare, aggressive non-Hodgkin lymphoma that is treated upfront mostly with L-asparaginase containing regimens. Relapsed extranodal natural killer/T-cell lymphoma is associated with a poor prognosis, and there is no established standard of care.

**Case presentation:**

We report the case of a 72 year-old white male with a distant extranasal relapse of extranodal natural killer/T-cell lymphoma that has been managed successfully with a combination of radiation and immune checkpoint blockade with pembrolizumab. Pseudoprogression with new skin and bone lesions on positron emission tomography imaging was encountered during this Caucasian patient’s immunotherapy and was successfully managed with supportive care and continuation of immune checkpoint blockade.

**Conclusions:**

The patient has been in complete clinical, radiologic, and molecular remission for close to 3 years and has not had any immune-related adverse effects. Pseudoprogression is a clinical challenge that can be encountered while patients are treated with immunotherapy, and astute clinical acumen is needed for accurate management. We believe this is the longest duration of response to immune checkpoint blockade in relapsed extranodal natural killer/T-cell lymphoma reported to date in literature. There is a strong biologic rationale in combining radiation with immunotherapy. The optimal timing, dose, and duration of radiation combined with immunotherapy in extranodal natural killer/T-cell lymphoma need to be prospectively evaluated.

## Background

Extranodal natural killer/T-cell lymphoma (ENKL) is an aggressive non-Hodgkin lymphoma known for frequent relapses and poor outcomes. Standard-of-care upfront therapy involves combined modality chemoradiation in limited stage disease, while combination chemotherapy with L-asparaginase containing regimens such as SMILE (dexamethasone, methotrexate, ifosfamide, L-asparaginase and etoposide) are used in advanced-stage disease, though treatment failure is seen in up to 40% of cases [1, 2]. The treatment needs of relapsed/refractory (R/R) ENKL remain unmet. Epstein–Barr virus (EBV)-infected cells are known to have increased PD-L1 expression, suggesting EBV-driven lymphomas may be specifically vulnerable to PD-1/PD-L1 blockade [3, 4]. There is a strong biologic rationale for combining radiation therapy with immune checkpoint blockade (iRT). We report a case with relapsed ENKL who achieved prolonged remission following iRT.

## Case presentation

**Fig. 1:**
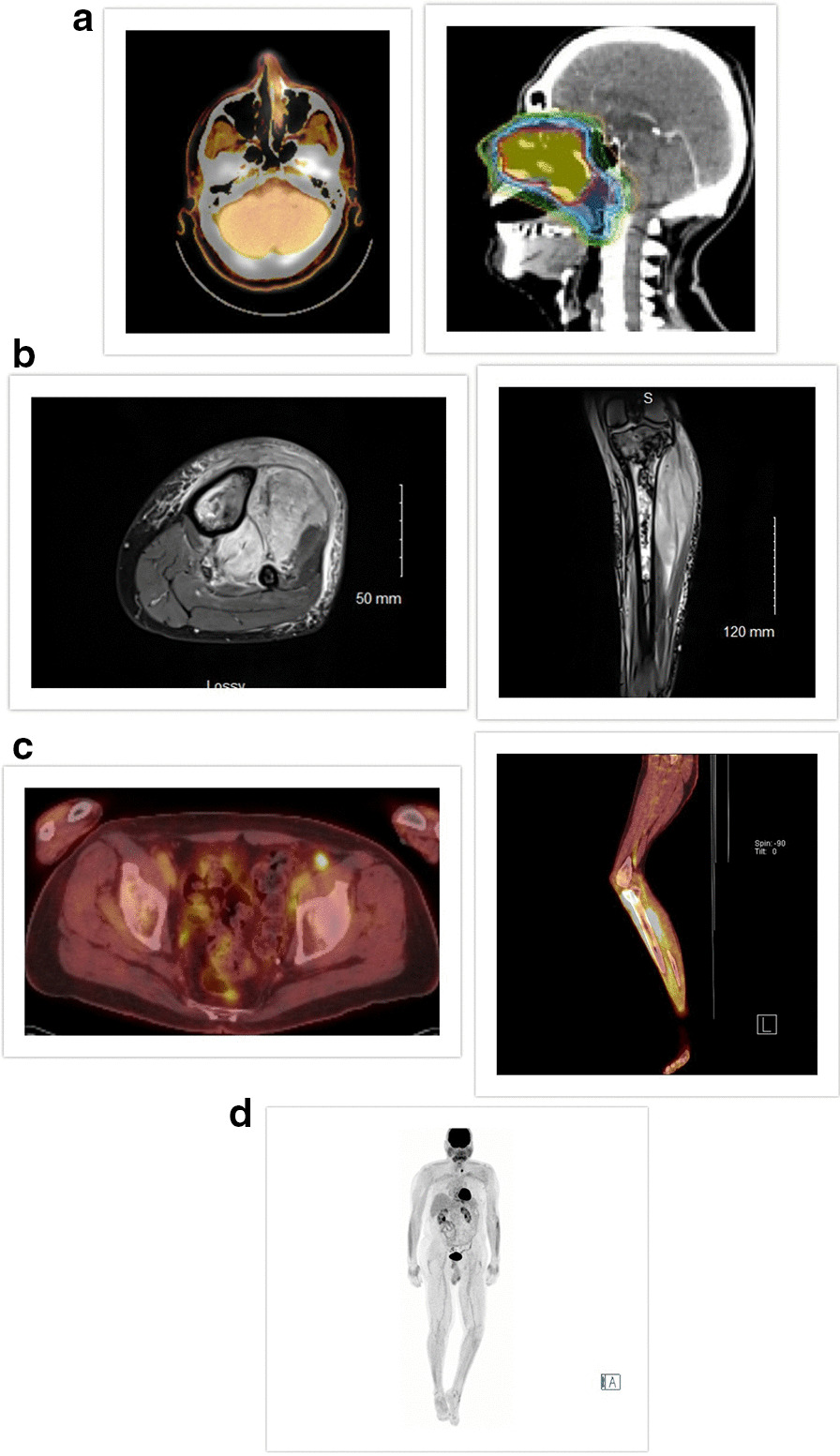
** a** Imaging showing “limited” stage I ENKL involving left nare/maxillary sinus at diagnosis and IMRT field used for therapy in 2013. **b** Magnetic Resonance Imaging of left lower extremity at relapse in 2018 showing cortical erosion of the left proximal tibia with marrow signal abnormality extending from the knee joint to the mid tibial diaphysis and prominent soft tissue abnormality of the musculature. **c** Positron Emission Tomography-Computed Tomography showing the left proximal pretibial mass measuring 9.4 × 9.7 cm (SUVmax 9.6) with FDG avid lymph nodes in the left inguinal and external iliac regions (SUVmax 4.82). **d** End-of-treatment Positron Emission Tomography- Computed Tomography showing evidence of complete metabolic remission

A 72-year-old white man with a history of coronary artery disease status/post coronary artery bypass graft, noninvasive urothelial carcinoma treated with transurethral resection without intravesical therapy, and localized prostatic adenocarcinoma on surveillance was diagnosed with “limited” stage IE ENKL, nasal-type involving the left nare/maxillary sinus in 2013. EBV titer was <1000 IU/mL at diagnosis. He was treated with SMILE for two cycles, though L-asparaginase was omitted after the first cycle because of transaminitis. He then completed consolidative chemoradiation with cisplatin to 45/50 Gy, using intensity modulated radiotherapy (IMRT) and two additional cycles of adjuvant chemotherapy (Fig. 1a). He achieved and remained in complete molecular and radiologic remission for over 4 years. In April 2018, he presented with left anterolateral leg pain, erythema, and swelling. Magnetic resonance imaging (MRI) showed left proximal tibial cortical erosion with marrow signal abnormality from the knee joint to the mid tibial diaphysis and prominent soft tissue abnormality involving the musculature. Positron emission tomography–computed tomography (PET-CT) showed the left proximal pretibial mass measuring 9.4 × 9.7 cm (SUVmax 9.6) with fluorodeoxyglucose (FDG) avid lymph nodes in the left inguinal and external iliac regions (SUVmax 4.82) (Fig. 1b, c).

**Fig. 2:**
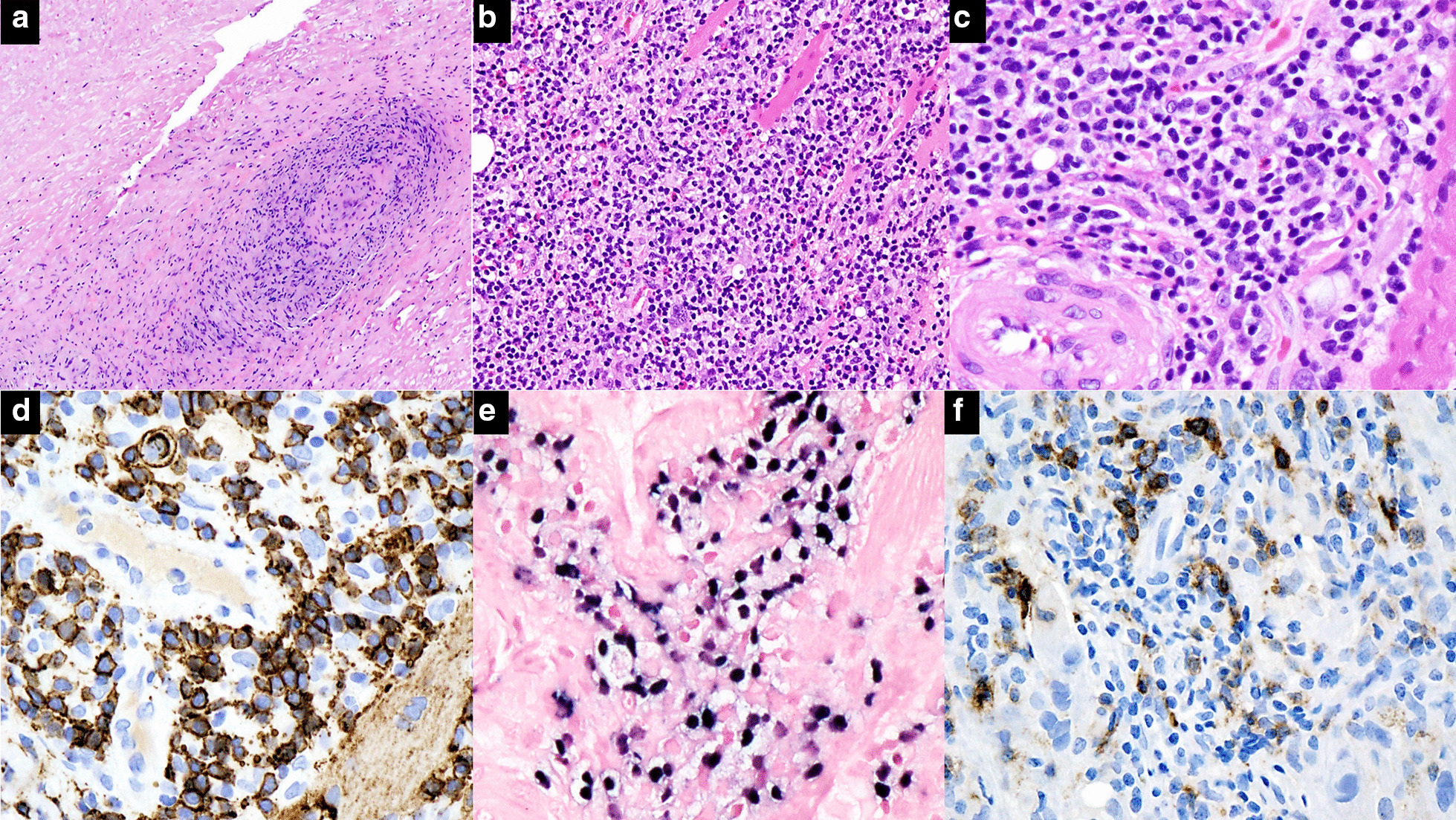
** a** Histologic sections containing extensive coagulative necrosis with angiodestruction and angioinvasion by an atypical lymphocytic infiltrate (hematoxylin and eosin, 4×). **b** Focally preserved areas showing invasion and destruction of skeletal muscle by an infiltrate of small-to-medium-sized atypical lymphocytes, eosinophils, and histiocytes (hematoxylin and eosin, 20×).** c** High-power view showing atypical lymphocytes with round to irregular nuclei and condensed chromatin admixed with scattered large cells (hematoxylin and eosin, 40×). **d** Atypical infiltrate showing strong expression of CD56 (see text for full immunophenotype) (CD56, 40×).** e** Positive *in situ* hybridization for EBV-encoded small RNA (EBER) in atypical cells (EBER, 40×). **f** Subset of cells showing expression of PD-1 (PD-1, 40×)

Morphologic examination of the left tibial biopsy showed extensive areas of coagulative necrosis, angioinvasion, and destruction. Focally preserved areas showed diffuse infiltrate of small-to-medium-sized atypical lymphocytes and scattered large lymphocytes with destruction of associated skeletal muscle. Immunohistochemistry showed atypical lymphocytes variably positive for CD2 and CD3 and negative for CD5, CD7, CD20, and CD68. The cells showed strong expression of CD56, TIA-1, and granzyme B. *In situ* hybridization for EBV-encoded small Ribonucleic acid (RNA) (EBER) was strongly positive. Immunohistochemistry for PD-1 was positive in a subset of atypical lymphocytes (Fig. 2a–f). Concurrent flow cytometry showed an aberrant cell population that was small to medium in size by forward scatter with the following immunophenotype: CD2(+), surface CD3(−), CD4(−), CD5(−), CD7(−), CD8(−), CD30(−), CD34(−), CD45( bright +), CD56(bright +), TCRαβ(−), and TCRγδ(−). Quantitative PCR for serum EBV was markedly elevated (17,072 IU/mL). PD-L1 expression by IHC (22C3 pharmDx, Dako) showed high expression with a tumor proportion score of 70%.

**Fig. 3:**
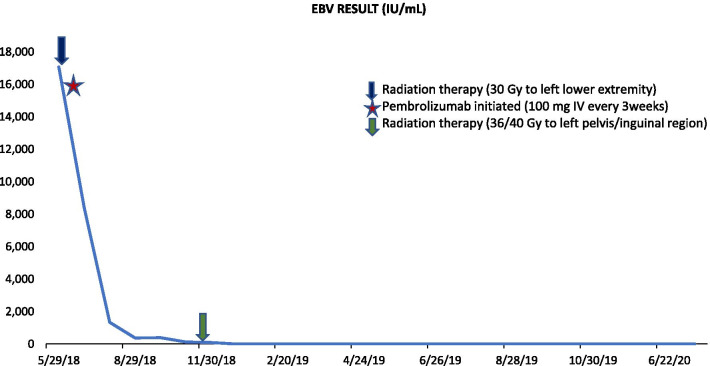
Graph showing temporal trend of Epstein-Barr virus (EBV) quantitative PCR in relation to radiation and immunotherapy

As he was symptomatic, he was initially treated with 30 Gy radiation over ten fractions to the left lower extremity followed by pembrolizumab (100 mg intravenously every 3 weeks). PET-CT after four cycles showed new FDG-avid lesions of the left foot and right proximal tibia but interval decrease in size and FDG avidity of the originally identified tibial lesion. The left foot lesion was biopsied, revealing EBV-negative, CD56-negative reactive lymphoid tissue. Given these biopsy findings representing “pseudoprogression,” pembrolizumab was continued. He then received consolidative radiation to the left pelvis and inguinofemoral basin, 36/40 Gy in 20 fractions. EBV quantitative PCR decreased rapidly and became undetectable after eight cycles (Fig. 3)*.* PET-CT showed continued decrease in FDG avidity of left leg anterior compartment consistent with response to treatment (Lugano 2-3) and no new FDG avid lesions [Fig. 1d). The patient has had no immune-related adverse events (irAEs) and has completed 40 cycles of pembrolizumab to date.

## Discussion and conclusions

Natural killer/T-cell lymphoma (NKTCL) is a rare disease with an incidence of 5.2% and 3% in Southeast Asia and South America, respectively, but only 0.3% in North America and Europe [3]. Median age at diagnosis is 52 years with a male predominance. It is typically extranodal and divided by primary site as nasal or extranasal [4]. Despite advances in therapy, extranasal NKTCL has a poor prognosis [5]. Clonal episomal EBV infection is integral to NK-cell lymphomagenesis, and its plasma DNA quantification has been used to assess burden of disease and response to treatment [6, 7].

There is no accepted standard of care in relapsed ENKL, with gemcitabine-based chemotherapy resulting in median progression-free survival of 2.3 months [8]. After the elucidation that PD-L1 expression is upregulated in EBV-infected cells, the use of anti-PD1 immune-checkpoint inhibitors has garnered attention in relapsed/refractory (R/R) ENKL [9]. Complete response with single agent pembrolizumab was seen in 71% patients with R/R disease in one retrospective cohort [10]. While all but one patient in this cohort received pembrolizumab 2 mg/kg every 3 weeks, our patient was treated with a 100 mg flat dose every 3 weeks based on a 2018 retrospective study showing similar efficacy [11, 12]. Similar results have also been shown with low-dose nivolumab [13]. Clinically significant irAEs reported in published case reports and series of anti-PD1 agents in ENKL include pneumonitis, grade 2 skin GVHD, myalgia, hypophosphatemia, thrombocytopenia, and cytokine release syndrome [14, 15]. Using whole-genome sequencing, clonal structural rearrangements of the *PD-L1* gene and *JAK3* activating mutations were demonstrated in a cohort of patients achieving a complete response to anti-PD1 therapy [16, 17].

The concept of “pseudoprogression” is clinically perilous. Pseudoprogression was described in two patients with relapsed ENKL on pembrolizumab; both had undetectable EBV quantitative PCR and new FDG-avid lesions that resolved with continued immunotherapy [10]. Biopsy of these lesions showed reactive lymphoid infiltrates composed primarily of CD3+ T cells without EBV+ or/and CD56+ cells, as in our case.

Published case series on the efficacy of checkpoint blockade in R/R ENKL report complete remission (CR) rates of 57–71% and durability of response ranging from 2 to 18 cycles [12, 13]. Our patient has been in an ongoing clinical, radiologic, and molecular complete remission on ICB therapy for 40 cycles (33 months). The combination of radiation with immunotherapy (iRT) has shown synergy in preclinical and clinical settings across tumor types. Immune stimulatory effects of radiation are due to multiple mechanisms including major histocompatibility complex (MHC) upregulation, release of damage associated molecular patterns [DAMPs such as calreticulin, adenosine triphosphate (ATP), high-mobility group box 1 (HMGB-1)] with cell death and reprogramming of the vascular and myeloid compartments of the tumor microenvironment. Though we cannot precisely discern the mechanism of this patient’s outlier response, it is reasonable to hypothesize that it may have been facilitated by the local debulking and/or abscopal effects of iRT [18].

Other therapies being explored in R/R ENKL include anti-CD30/CD38 monoclonal antibodies, JAK-STAT, PI-3 kinase, and proteasome inhibitors, EBV cytotoxic T cells, and cytokine modulation [19, 20]. Allogeneic stem cell transplantation on retrospective registry analysis has shown long-term survival in only about one-third of patients [21].

This report shows a patient with relapsed extranasal ENKL successfully treated with radiation and pembrolizumab. To our knowledge, this is the longest ongoing progression-free survival in relapsed ENKL without undergoing allogeneic stem cell transplantation. The appropriate timing, dose, and duration of radiation and immune checkpoint blockade in treating ENKL need to be prospectively studied.

## Data Availability

Data sharing not applicable to this article as no datasets were generated or analyzed during the current study.

## Abbreviations

ENKL: Extranodal natural killer/T-cell lymphoma
R/R: Relapsed/refractory
iRT: Combined immunoradiotherapy
SMILE: Chemotherapy regimen containing steroids (dexamethasone), methotrexate, ifosfamide, L-asparaginase, and etoposide
ICB: Immune checkpoint blockade
PD1: Programmed cell death protein 1
IMRT: Intensity modulated radiation therapy
EBV: Epstein–Barr virus
SUV: Standardized uptake value
FDG: Fluorodeoxyglucose
